# Associations of the *FTO* rs9939609 and the *MC4R* rs17782313 polymorphisms with type 2 diabetes are modulated by diet, being higher when adherence to the Mediterranean diet pattern is low

**DOI:** 10.1186/1475-2840-11-137

**Published:** 2012-11-06

**Authors:** Carolina Ortega-Azorín, Jose V Sorlí, Eva M Asensio, Oscar Coltell, Miguel Ángel Martínez-González, Jordi Salas-Salvadó, Maria-Isabel Covas, Fernando Arós, José Lapetra, Lluís Serra-Majem, Enrique Gómez-Gracia, Miquel Fiol, Guillermo Sáez-Tormo, Xavier Pintó, Miguel Angel Muñoz, Emilio Ros, Jose M Ordovás, Ramon Estruch, Dolores Corella

**Affiliations:** 1 Department of Preventive Medicine and Public Health, School of Medicine, University of Valencia, Valencia, Spain; 2 CIBER Fisiopatología de la Obesidad y Nutrición, Instituto de Salud Carlos III, Madrid, Spain; 3 Department of Computing Languages and Systems, University Jaume I, Castellon, Spain; 4 Nutrition and Genomics Laboratory, JM-USDA Human Nutrition Research Center on Aging at Tufts University, Boston, MA, USA; 5 Department of Preventive Medicine and Public Health, School of Medicine, University of Navarra, Pamplona, Spain; 6 Human Nutrition Unit, Faculty of Medicine, IISPV, University Rovira i Virgili, Reus, Spain; 7 Cardiovascular Epidemiology Unit, Municipal Institut for Medical Research (IMIM), Barcelona, Spain; 8 Department of Cardiology, Hospital Txagorritxu, Vitoria, Spain; 9 Department of Family Medicine, Primary Care Division of Sevilla, San Pablo Health Center, Sevilla, Spain; 10 Department of Clinical Sciences, University of Las Palmas de Gran Canaria, Las Palmas de Gran Canaria, Spain; 11 Department of Epidemiology, School of Medicine, University of Malaga, Málaga, Spain; 12 University Institute for Health Sciences Investigation, Hospital Son Dureta, Palma de Mallorca, Spain; 13 Department of Biochemistry, School of Medicine, University of Valencia, Valencia, Spain; 14 Lipids and Vascular Risk Unit, Internal Medicine, Hospital Universitario de Bellvitge, Hospitalet de Llobregat, Barcelona, Spain; 15 Primary Care Division, Catalan Institute of Health, Barcelona, Spain; 16 Lipid Clinic, Endocrinology and Nutrition Service, Institut d’Investigacions Biomèdiques August Pi Sunyer (IDIBAPS), Hospital Clinic, Barcelona, Spain; 17 Department of Cardiovascular Epidemiology and Population Genetics, Centro Nacional de Investigaciones Cardiovasculares (CNIC), Madrid, Spain; 18 IMDEA Alimentación, Madrid, Spain; 19 Department of Internal Medicine, Hospital Clinic, IDIBAPS, Barcelona, Spain; 20 Genetic and Molecular Epidemiology Unit, Valencia University, Blasco Ibañez, 15, 46010, Valencia, Spain

**Keywords:** Nutrigenetics, Mediterranean diet, Diabetes, FTO, MC4R, Gene-diet interactions

## Abstract

**Background:**

Although the Fat Mass and Obesity (*FTO*) and Melanocortin-4 Receptor (*MC4R*) genes have been consistently associated with obesity risk, the association between the obesity-risk alleles with type 2 diabetes is still controversial. In some recent meta-analyses in which significant results have been reported, the associations disappeared after adjustment for body mass index (BMI). However gene-diet interactions with dietary patterns have not been investigated. Our main aim was to analyze whether these associations are modulated by the level of adherence to the Mediterranean Diet (MedDiet).

**Methods:**

Case-control study in 7,052 high cardiovascular risk subjects (3,430 type 2 diabetes cases and 3,622 non-diabetic subjects) with no differences in BMI. Diet was assessed by validated questionnaires. *FTO*-rs9939609 and *MC4R*-rs17782313 were determined. An aggregate genetic score was calculated to test additive effects. Gene-diet interactions were analyzed.

**Results:**

Neither of the polymorphisms was associated with type 2 diabetes in the whole population. However, we found consistent gene-diet interactions with adherence to the MedDiet both for the *FTO-*rs9939609 (P-interaction=0.039), the *MC4R*-rs17782313 (P-interaction=0.009) and for their aggregate score (P-interaction=0.006). When adherence to the MedDiet was low, carriers of the variant alleles had higher type 2 diabetes risk (OR=1.21, 95%CI: 1.03-1.40; P=0.019 for *FTO-*rs9939609 and OR=1.17, 95%CI:1.01-1.36; P=0.035 for *MC4R*-rs17782313) than wild-type subjects. However, when adherence to the MedDiet was high, these associations disappeared (OR=0.97, 95%CI: 0.85-1.16; P=0.673 for *FTO-*rs9939609 and OR=0.89, 95%CI:0.78-1.02; P=0.097 for *MC4R*-rs17782313). These gene-diet interactions remained significant even after adjustment for BMI. As MedDiet is rich in folate, we also specifically examined folate intake and detected statistically significant interaction effects on fasting plasma glucose concentrations in non-diabetic subjects. However these findings should be interpreted with caution because folate intake may simply reflect a healthy dietary pattern.

**Conclusions:**

These novel results suggest that the association of the *FTO*-rs9939609 and the *MC4R*-rs17782313 polymorphisms with type 2 diabetes depends on diet and that a high adherence to the MedDiet counteracts the genetic predisposition.

## Background

The Fat Mass and Obesity (*FTO*) and Melanocortin-4 Receptor (*MC4R*) genes are considered leading obesity-associated loci [1-6]. Both genes have been found to be highly expressed in the hypothalamus in rats [7] suggesting a role in a role in the central regulation of energy balance and appetite [7,8]. Although common variations in these genes have been consistently associated with a higher body mass index (BMI) and obesity risk in numerous individual studies and meta-analyses [1-6,9-12], the association of these variants with a higher type 2 diabetes risk has only recently come to the fore, and is still highly controversial [9,13-24]. Regarding the *FTO* gene, a recent meta-analysis [13] in East and South Asians, concluded that the *FTO* rs9939609 minor allele (or a proxy), the risk allele for obesity, increased the risk of type 2 diabetes, this association remaining statistically significant even after adjustment for BMI. Similar results were reported in a Scandinavian population [14]. In contrast, other studies, despite finding a higher type 2 diabetes risk in carriers of the risk-allele for obesity, concluded that this association disappears when adjusting for BMI [1,25-27]. There are also several investigations in which no association with type 2 diabetes was found [6,9,28-30]. Along these lines, a recent editorial comment [24] stated that it is still unclear whether the *FTO* is a diabetes-susceptibility gene and further data are needed at this stage, recommending that, in future studies, cases of type 2 diabetes and controls be paired by BMI to better analyze the independent effects on the two outcomes. Regarding the *MC4R* gene, there are fewer studies that have analyzed the association between the rs17782313 polymorphism (or a proxy) with type 2 diabetes than for the *FTO* gene, and the results are even less conclusive [2,18,19,22,23,25,31].

Despite the numerous studies carried out in various populations, it is surprising that none of the above mentioned [1-31] have specifically examined the influence of the diet modulating the associations of the *FTO* and the *MC4*R risk alleles with type 2 diabetes. The analysis of this modulation is of great importance as it has been reported that mice with increased *fto* expression did not develop glucose intolerance when fed a standard diet [32]. Interestingly, they developed glucose intolerance on a high fat diet [32]. Likewise, *MC4R* knockout mice exhibited increased adiposity and hyperinsulinemia and sometimes, depending on diet, developed type 2 diabetes [33]. Every day more importance is placed on the overall food intake pattern on type 2 diabetes [34-36] and the traditional MedDiet pattern, low in saturated fat and rich in vegetables, fruits, legumes, fish, nuts and olive oil, reduces type 2 diabetes incidence [36-38]. Then, outstanding among the dietary factors that could modulate the effect of the *FTO* rs9939609 and the *MC4R* rs17782313 polymorphisms on type 2 diabetes, is the Mediterranean diet (MedDiet). Furthermore, the MedDiet is rich in folates [39] and folate availability is crucial for DNA methylation status [40]. Considering that dysregulation in DNA, methylation has been suggested as one relevant epigenetic mechanism in type 2 diabetes [41] and that both the *FTO*[42] and the *MC4R*[43] genes are regulated by methylation, the MedDiet might modulate the effect of these genes through epigenetic mechanisms.

As there are no published studies either for the *MC4R* or for the *FTO* genes that have analyzed their interactions with MedDiet on type 2 diabetes, our main objective was to evaluate whether adherence to the MedDiet pattern modifies the association of the *FTO* rs9939609 and *MC4R* rs17782313 polymorphisms with type 2 diabetes, either independently or jointly. Our secondary aim was to examine the contribution of folate intake in this interaction.

## Methods

### Subjects

In a case-control study, we analyzed 7,052 participants (3,430 cases with type 2 diabetes and 3,622 non-diabetic controls) from the PREDIMED (PREvención con DIeta MEDiterránea) trial from whom DNA was isolated, the *FTO* rs9939609 determined, and who had valid data for the main clinical and lifestyle variables analyzed at baseline. These participants did not differ in the main characteristics from those of the total cohort (n=7,447). In 7,019 of them, the *MC4R* rs17782313 polymorphism was successfully determined. The PREDIMED study (http://www.predimed.org) is a multi-center clinical trial aimed at assessing the effects of the MedDiet on the primary prevention of cardiovascular disease (CVD) [44,45]. Participants were recruited between 2003 and 2009 in Primary Care Centers affiliated to 11 recruiting centers (teaching Hospitals) in Spain. They were women (60 to 80 years) or men (55 to 80 years) without prior CVD, with type 2 diabetes (cases) or at least three of the following cardiovascular risk factors in subjects without type 2 diabetes (controls): current smoking, hypertension, elevated low-density lipoprotein cholesterol, low high-density lipoprotein cholesterol, overweight/obesity, or family history of premature coronary heart disease. The specific cut-off points for these eligibility criteria have been previously described [45]. Type 2 diabetes was diagnosed according to American Diabetes Association criteria [46]. The duration of type 2 diabetes was also recorded. The Institutional Review Board /Ethics Committee of each participating center approved the study protocol. All participants provided written informed consent. The study is registered at http://www.controlled-trials.com/ISRCTN 35739639.

### Clinical, anthropometric and dietary measurements

A general questionnaire was administered at baseline as previously reported [45]. Weight and height were directly measured with calibrated scales and a wall-mounted stadiometer, respectively. Body mass index (BMI) was calculated as weight in kilograms divided by the square of height in meters [45]. Registered dietitians completed a validated 14-item MedDiet adherence questionnaire in a face-to-face interview with each participant [47]. This questionnaire consists of 14 questions on the frequency of consumption of specific foods characteristic of the Spanish MedDiet. Each question was scored 0 or 1. One point was given for: 1) using olive oil as the principal source of fat for cooking; 2) preferring white meat over red meat, or for consuming: 1) 4 or more tablespoons of olive oil/d; 2) 2 or more servings of vegetables/d; 3) 3 or more pieces of fruit/d; 4) <1 serving of red meat or sausages/d; 5) <1 serving of animal fat/d; 6) <1 cup of sugar-sweetened beverages/d; 7) 7 or more servings of red wine/wk; 8) 3 or more servings of pulses/wk; 9) 3 or more servings of fish/wk; 10) fewer than 2 commercial pastries/wk; 11) 3 or more servings of nuts/wk; or 12) 2 or more servings/wk of a dish with a traditional sauce of tomatoes, garlic, onion, or leeks sautéed in olive oil. If the condition was not met, 0 points were recorded for the category. The final score ranged from 0 to 14 points. The greater the score obtained from the questionnaire, the greater the adherence to the MedDiet. A dichotomous variable of adherence to the MedDiet was created using as cut-off points the sample mean. In a recent work [48] we have demonstrated the important association of the scores obtained in this questionnaire with obesity phenotypes reinforcing the observation that it is a valid tool that may have a great impact on the genotype-phenotype relationship.

In addition, a 137-item validated food frequency questionnaire [49] was administered to all participants. Energy and nutrient intake were calculated from Spanish food composition tables [50]. Dichotomous variables for nutrient intake were also created using as cut-off points the sample means. Physical activity was estimated by the Minnesota Leisure Time Physical Activity Questionnaire validated in Spain [51].

### Biochemical analysis, DNA extraction and genotyping

Blood samples were obtained after an overnight fast. Fasting glucose was measured using standard enzymatic automated methods as previously described [44].

Genomic DNA was extracted from buffy-coat with the MagNaPure LC DNA Isolation Kit (Roche Diagnostics, Mannheim, Germany). The *MC4R* rs17782313 and *FTO* rs9939609 polymorphisms were genotyped on a 7900HT Sequence Detection System (Applied Biosystems, FosterCity, CA, USA) using fluorescent allelic discrimination TaqManTM assays. The calling rate for both polymorphisms was >95%. For quality control purposes, 5% of samples were randomly selected and genotyped a second time. There were no discrepancies between the two results. Genotype frequencies did not deviate from Hardy-Weinberg equilibrium expectations for either polymorphism (P=0.709 for the *FTO* rs9939609 and P= 0.637 for the *MC4R* rs17782313).

### Statistical analysis

Genetic variables were tested using dominant models of the *FTO* rs9939609 and the *MC4R* rs17782313 polymorphisms individually. Also, an additive genetic score was created from the two polymorphisms in which the presence of each of the variant alleles for each polymorphism was scored as one point. The range of values of this aggregate score variable varied from 0 to 4 points. As the number of subjects with a score of 4 points was very low, a new score variable (score-grouped) was created grouping the categories of 3 and 4 points. Chi-square tests were used to analyze differences between observed and expected genotype frequencies, assuming Hardy–Weinberg equilibrium, and to test differences in percentages. We used t-test and ANOVA to compare crude means of continuous variables. Multivariate adjustments for comparisons of continuous variables were carried out by generalized linear models. Multivariable logistic regression methods were used to estimate the odds ratios (OR) of the *MC4R* or *FTO* polymorphisms and type 2 diabetes and to adjust for confounders. Models were first adjusted for age, sex and center. Additional adjustments for BMI, total energy intake, physical activity, adherence to the MedDiet, tobacco smoking, alcohol consumption or education were also carried out as indicated. Dichotomous variables for dietary intake and physical activity were created using as cut-off the sample means. The homogeneity of the effects by sex was also statistically tested using the likelihood ratio test. To examine the interaction between the *MC4R* rs17782313, the *FTO* rs9939609 polymorphisms or their score and adherence to the MedDiet, or the other dietary variables, we fitted separate multivariate regression models including the corresponding main effects and interaction terms in addition to the potential confounders. The likelihood ratio test was used to obtain the P values for interactions. Stratified analyses were also carried out. Statistical analyses were performed with the SPSS package, version 15.0 (SPSS, Chicago, IL). All tests were two-tailed and P values <0.05 were considered statistically significant.

## Results

We studied 3,430 subjects with type 2 diabetes and 3,622 non-diabetic subjects (57% women, mean age 70+/-7 years). Because of the selection criteria, type 2 diabetes cases did not have higher BMI than non-diabetic subjects. Table 1 shows the demographic, biochemical, clinical, lifestyle and genetic characteristics of these participants depending on diabetes status. For the whole sample, mean (±SD) adherence to the MedDiet was 9±2 points on the scale of 0 to 14. We found a small but statistically significant difference (P=0.003) in the mean adherence to the MedDiet depending on the type 2 diabetes status. Duration of type 2 diabetes was as follows: <1 year post-diagnosis (10% of diabetic subjects), 1-5 years (40%) and more than 5 years post-diagnosis (50%). We did not observe significant differences in the mean of adherence to the MedDiet depending on the duration of diabetes (P=0.227), indicating that the pattern of adherence to the MedDiet remains stable regardless of the diabetes diagnostic.

**Table 1:** Demographic, clinical, lifestyle and genetic characteristics of the study participants at baseline

	**Total**		**No type 2 diabetes**		**Type 2 diabetes**		
	**(n=7,052)**		**(n=3,622)**		**(n=3,430)**		
	**Mean**	**(SD)**	**Mean**	**(SD)**	**Mean**	**(SD)**	**P**
Men/women, n	3008/4044		1382/2240		1626/1804		<0.001
Age (years)	66.9	(6.2)	66.6	(6.1)	67.3	(6.2)	<0.001
Weight (kg)	76.8	(11.9)	76.6	(11.7)	76.9	(12.2)	0.378
BMI (kg/m^2^)	29.9	(3.8)	30.0	(3.7)	29.9	(4.0)	0.066
Waist circumference (cm)	100.4	(10.6)	99.7	(10.6)	101.2	(10.5)	<0.001
Adherence to the Mediterranean diet	8.7	(1.9)	8.7	(2.1)	8.5	(1.9)	0.003
Energy intake (kcal/d)	2276	(607)	2322	(604)	2228	(607)	<0.001
Total fat (g/d)	98.7	(30.4)	99.1	(29.6)	98.6	(31.3)	0.534
Saturated fat (g/d)	25.4	(9.2)	25.2	(9.0)	25.5	(9.4)	0.155
MUFA (g/d)	48.8	(16.1)	49.2	(15.5)	48.6	(16.6)	0.680
PUFA (g/d)	15.9	(7.0)	15.8	(7.2)	15.9	(7.2)	0.816
Carbohydrates (g/d)	239	(81)	250	(82)	229	(78)	<0.001
Fiber (g/d)	25.7	(9.2)	25.9	(9.3)	25.4	(9.1)	0.063
Alcohol consumption (g/d)	8.4	(14.1)	9.1	(14.7)	7.6	(13.4)	<0.001
Folic acid (microg/d)	406	(127)	407	(125)	406	(129)	0.777
Physical activity* (kcal/d)	230	(239)	225	(226)	237	(253)	0.028
Fasting glucose (mg/dL)**	122.2	(41.0)	98.2	(16.4)	147.3	(45.0)	<0.001
Current smokers (%)	14.1		16.0		12.1		0.001
Obesity (%)	46.7		47.0		46.4		0.595
Genotypes (%)							
*FTO* rs9939609							
TT	33.0		33.9		32.1		0.227
TA	48.7		48.3		49.1		
AA	18.3		17.8		18.8		
*MC4R* rs17782313***							
TT	61.8		61.9		61.9		0.965
TC	33.5		33.4		33.4		
CC	4.7		4.7		4.7		

P: P-value for the comparison between subjects with type 2 diabetes and non-diabetes.

*Leisure time physical activity; **: Fasting glucose data were available for 6232 participants.

***Genotype data for the *MC4R* were obtained for 7019 subjects.

BMI: Body mass index; MUFA: Monounsaturated fatty acids; PUFA: polyunsaturated fatty acids.

We did not observe statistically significant differences in genotype frequencies of the *FTO* rs9939609 or the *MC4R* rs17782313 polymorphisms between subjects with type 2 diabetes and non-diabetic subjects. The aggregate score of these two polymorphisms had a prevalence of 20.6% for zero points (homozygous subjects for non-variant alleles); 40.9% for 1 point (subjects with one variant allele either at *FTO* or *MC4R*; 29.2% for 2 points (subjects with two variant alleles), 8.5% for 3 points (subjects with 3 variant alleles) and 0.8% for 4 points (homozygous subjects for the variant alleles both at the *FTO* and *MC4R* genes) in the whole sample. We did not detect significant differences for the aggregate score by diabetes status (P=0.640). The *FTO* polymorphism was significantly associated with higher BMI in carriers of the variant allele (30.1± 3.9 in TA+AA subjects *vs* 29.8±3.8 kg/m^2^ in TT; P=0.043), whereas the effect of the *MC4R* polymorphism did not reach the statistical significance (30.1± 3.9 in TC+CC subjects *vs* 29.9±3.8 kg/m^2^ in TT; P=0.187). Likewise, the *FTO* polymorphism was significantly associated with waist circumference (100.7± 10.4 in TA+AA subjects *vs* 99.9±10.8 cm in TT; P=0.008) and non-significant differences were observed for the *MC4R*, although a similar trend was found 100.7± 11.0 in TC+CC subjects *vs* 100.3±10.4 cm in TT); P=0.137.

### Association between the FTO rs9939609 and MC4R rs17782313 polymorphisms and type 2 diabetes

We did not find (Table 2) any statistically significant association between the *FTO* rs9939609 polymorphism and type 2 diabetes when analyzing the population as a whole (OR:1.07, 95%CI: 0.97-1.18; P=0.191 for carriers of the *FTO* obesity risk allele in comparisons with TT homozygotes in model 2). Additional adjustment for BMI did not change the results (model 3). Likewise, there was no association between the *MC4R* rs17782313 polymorphism and type 2 diabetes in the whole sample (OR:1.01, 95%CI: 0.92-1.12; P=0.837 for carriers of the *MC4R* risk allele in comparison with TT homozygotes. Neither was the aggregate score associated with type 2 diabetes (P=0.572). Additional adjustment for BMI did not change the associations. We observed no heterogeneity by gender (P for interactions >0.05).

**Table 2:** **Association between the *FTO*, the *MC4R *polymorphisms and the combined score (*FTO *and *MC4R*) and type 2 diabetes**

	**Model 1**		**Model 2**		**Model 3**	
**Genetic variants**	**OR**	**95% CI**	**OR**	**95% CI**	**OR**	**95% CI**
***FTO*rs9939609** (n=7,052)						
*Genotypes*						
TT	1.00	(reference)	1.00	(reference)	1.00	(reference)
TA+AA	1.08	(0.97-1.19)	1.07	(0.97-1.18)	1.07	(0.97-1.19)
	P=0.147		P=0.191		P=0.181	
Variant allele effects**						
(Per A allele)	1.06	(0.99-1.14)	1.06	(0.98-1.13)	1.06	(0.98-1.13)
	P=0.079		P=0.118		P=0.111	
***MC4*R rs17782313** (n=7,019)						
TT	1.00	(reference)	1.00	(reference)	1.00	(reference)
TC+CC	1.01	(0.92-1.12)	1.01	(0.92-1.12)	1.01	(0.91-1.12)
	P=0.832		P=0.837		P=0.845	
Variant allele effects**						
(Per C allele)	1.01	(0.93-1.10)	1.01	(0.93-1.09)	1.01	(0.93-1.09)
	P=0.808		P=0.817		P=0.800	
**Aggregate score (*FTO*/*MC4R*)**						
TT and TT (0)	1.00	(reference)	1.00	(reference)	1.00	(reference)
TA or TC (1)	1.06	(0.93-1.21)	1.06	(0.93-1.20)	1.06	(0.93-1.20)
TA and TC or AA or CC (2)	1.10	(0.96-1.27)	1.10	(0.96-1.27)	1.10	(0.96-1.27)
Otherwise (3 or 4 variants)	1.11	(0.92-1.34)	1.10	(0.90-1.32)	1.10	(0.91-1.33)
	P=0.553		P=0.572		P=0.553	
Variant allele effects, score***						
(Per variant allele: 1,2,3,or 4)	1.04	(0.99-1.10)	1.04	(0.98-1.10)	1.04	(0.98-1.09)
	P=0.137		P=0.183		P=0.169	

Multivariate logistic Regression analysis.

Model 1: Adjusted for sex, age and center.

Model 2: Adjusted for sex, age, center, total energy intake and physical activity.

Model 3: Adjusted for sex, age, center, total energy intake, physical activity and BMI.

**: A variable indicating the number of variant alleles (0, 1 or 2) was created for both the FTO and the *MC4R.*

***: For the estimation as a score, a variable indicating the number of combined variant alleles was created (0, 1, 2, 3 or 4).

P: P value obtained for the global effect of the polymorphism in the multivariate logistic regression models.

### Gene-diet interaction between the FTO rs9939609 and MC4R rs17782313 polymorphisms and adherence to the MedDiet in determining type 2 diabetes

We found a relevant interaction between adherence to the MedDiet and these polymorphisms in determining type 2 diabetes (Table 3), which was significant both for the *FTO* (P-interaction=0.039) and for the *MC4R* (P-interaction=0.009) as well as for their aggregate score (P-interaction=0.006) (Model 1). According to these interactions, the association or not of these polymorphisms with type 2 diabetes depended on the degree of adherence to the MedDiet. When adherence to the MedDiet was low (=<9 points), carriers of the variant allele (obesity-risk allele) had a higher risk of prevalent type 2 diabetes (OR=1.21, 95%CI: 1.03-1.40; P=0.019 for *FTO* and OR=1.17, 95%CI:1.01-1-36; P=0.035 for *MC4R*) than homozygous subjects for the major allele. However, when adherence to the MedDiet was high (>=9 points), there was no association of these polymorphisms with type 2 diabetes (OR=0.97, 95%CI: 0.85-1.16; P=0.673 for the *FTO* and OR=0.89, 95%:0.78-1-02; P=0.097 for the *MC4R*). These interactions remained statistically significant even after adjustment for BMI (P-interaction= 0.039 for *FTO*, P-int=0.009 for *MC4R* and P=0.006 for the aggregate score) (Model 2). Further adjustments for alcohol, tobacco smoking or education did not change the statistical significance of the results (not shown). These polymorphisms had an additive effect in the interaction with MedDiet on type 2 diabetes. So, when the aggregate score was considered as a continuous variable, we also obtained a statistically significant interaction effect (P-interaction=0.024 after adjustment for BMI). When we considered the aggregate genetic score (score-grouped) as a categorical variable, individuals carrying 3 or 4 variant alleles for the *FTO* rs9939609 and the *MC4R* rs17782313 polymorphisms had 45% higher odds (P=0.009) of prevalent type 2 diabetes (OR: 1.45; 95%CI:1.09-1.92) than subjects with no risk alleles if their adherence to the MedDiet was low. However, when adherence to the MedDiet was high, the higher risk of type 2 diabetes in subjects carrying variant alleles at both the *FTO* and the *MC4R* loci, were completely blunted (OR: 0.86; P=0.266).

**Table 3:** **Association between the *FTO, the MC4R *and the combined score (*FTO *and *MC4R *polymorphisms) and type 2 diabetes**

	**Model 1**					**Model 2**				
	**Adherence to the Mediterranean diet**					**Adherence to the Mediterranean diet**				
	**Low (<9 points)**		**High (>=9 points)**		**P**^**2**^**interaction**	**Low (<9 points)**		**High (>=9 points)**		**P**^**2**^**interaction**
	**OR**	**95% CI**	**OR**	**95% CI**	**Gene x AMD**	**OR**	**95% CI**	**OR**	**95% CI**	**Gene x AMD**
***FTO*rs9939609** (n=7,052)					0.039					0.039
TT	1.00	(reference)	1.00	(reference)		1.00	(reference)	1.00	(reference)	
TA + AA	1.21	(1.03-1.40)	0.97	(0.85-1.13)		1.20	(1.03-1.40)	0.97	(0.85-1.12)	
	P^1^=0.019		P^1^=0.673			P^1^=0.020		P^1^=0.743		
**MC4R rs17782313** (n=7,019)					0.009					0.009
TT	1.00	(reference)	1.00	(reference)		1.00	(reference)	1.00	(reference)	
TC + CC	1.17	(1.01-1.36)	0.89	(0.78-1.02)		1.17	(1.01-1.36)	0.89	(0.78-1.02)	
	P^1^=0.035		P^1^=0.097			P^1^=0.036		P^1^=0.102		
**Aggregate score (*FTO*/*MC4R*)**					0.006					0.006
TT and TT (0)	1.00	(reference)	1.00	(reference)		1.00	(reference)	1.00	(reference)	
TA or TC (1)	1.26	(1.05-1.56)	0.89	(0.75-1.07)		1.27	(1.06-1.56)	0.89	(0.75-1.07)	
TA and TC or AA or CC (2)	1.29	(1.05-1.59)	0.96	(0.79-1.16)		1.29	(1.05-1.59)	0.96	(0.79-1.17)	
Otherwise (3 or 4 variants)	1.45	(1.10-1.93)	0.86	(0.66-1.12)		1.45	(1.09-1.92)	0.87	(0.69-1.13)	
	P^1^=0.024		P^1^=0.532			P^1^=0.024		P^1^=0.513		
Variant allele effects**					0.012					0.012
(Per variant allele: 1,2,3,or 4)	1.12	(1.03-1.21)	0.97	(0.91-1.05)		1.12	(1.03-1.21)	0.97	(0.91-1.05)	
	P^1^=0.005		P^1^=0.475			P^1^=0.006		P^1^=0.532		

Stratified multivariate* logistic regression analysis depending on the adherence to the Mediterranean diet (AMD).

*: Models adjusted for sex, age, center, total energy intake and physical activity (Model 1). Model 2 was additionally adjusted for BMI.

**: For the estimation of the variant allele effect, the score variable indicating the number of combined variant alleles (0, 1, 2 , 3 and 4) was considered as continuous.

P1: P value obtained for the global effect of the polymorphism in the multivariate logistic regression models.

P2: P value for the interaction term between adherence to the Mediterranean diet and the corresponding polymorphism in the logistic regression model.

Furthermore, we adjusted the interaction models for waist circumference instead of BMI. This adjustment in the multivariate model (Model 2) did no change the level of significance of the interactions terms between the polymorphisms and adherence to the Mediterranean diet in determining type 2 diabetes (P-int: 0.034 for *FTO*, P-int: 0.010 for *MC4R* and P-int: 0.015 for the aggregate score). Thus, this gene-diet interaction remained statistically significant even adjustment for waist circumference.

### Gene-diet interactions between the FTO rs9939609 and MC4R rs17782313 polymorphisms and folate intake on type 2 diabetes and fasting glucose concentrations

**Figure 1:**
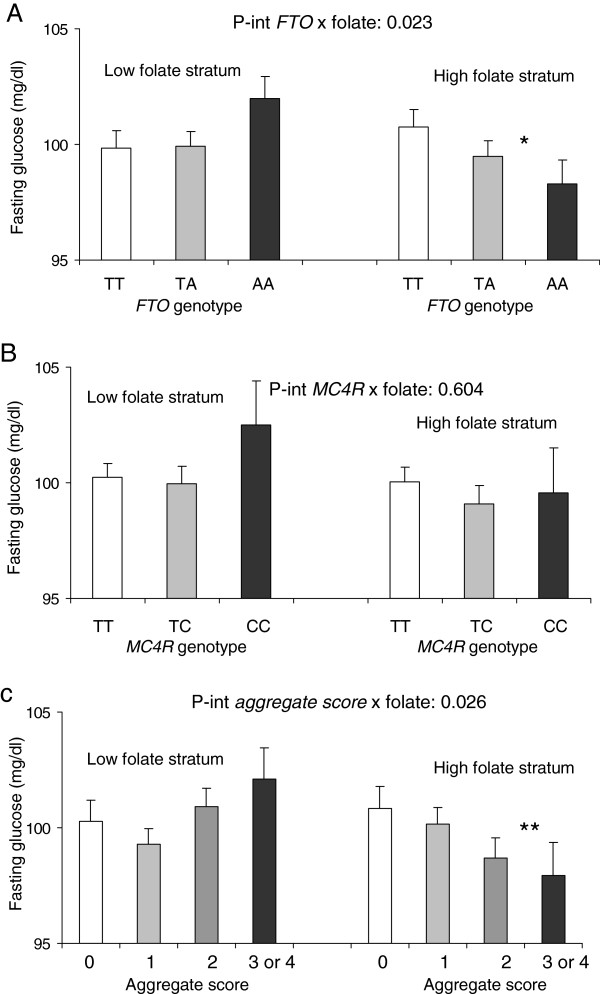
**Adjusted means of fasting glucose concentrations depending on the *FTO *rs9939609 (A), MC4R rs17782313 (B) or their grouped aggregate score (C) and the level of folate intake (low <406μg/d or high ≥406μg/d) in non-diabetic subjects (n=3192 for the *FTO *rs9939609 and n=3180 for the *MC4R *rs17782313.** P-int: P interaction values for the corresponding interaction terms between folate intake (as dichotomous) and the genetic variant obtained in the corresponding multivariate adjusted regression model including sex, age (as continuous), center, total energy intake (as continuous), physical activity (as dichotomous), folate intake (as dichotomous), the genetic variable (as categorical), and body mass index (as continuous) as covariates. *: P=0.043 for trend in the comparison of means in the multivariate adjusted model depending on the *FTO* rs9939609 genotype; **: P=0.027 for trend in the comparison of means in the adjusted model depending on the *FTO/MC4R* aggregate score. Error bars: SE of means.

We analyzed the interaction between folate intake (as dichotomous based on the population mean of 406 μg/d) and the polymorphisms on type 2 diabetes, but we did not obtain any statistically significant interaction term (P-interaction=0.203 for the *FTO* rs9939609, P-interaction=0.745 for the *MC4R* rs17782313 and P-interaction=0.667). As changes in methylation are very dynamic, we hypothesized a more direct effect of folate intake on fasting glucose concentrations in non-diabetic subjects, as diabetic subjects were taking medication and this could alter the results. We found (Figure 1) a statistically significant interaction (P=0.023) between the *FTO* rs9939609 polymorphism and folate intake on fasting glucose concentrations in non-diabetic subjects (Figure 1A). Thus, when folate intake was low, carriers of the variant allele had higher fasting plasma glucose concentrations than wild-type subjects. However, this was not observed when folate intake was high. Although, for the *MC4R* rs17782313, we found no significant interaction (Figure 1B), on analyzing the joint variable of both polymorphisms, the interaction term reached statistical significance (P=0.026) (Figure 1C). After adjustment of the multivariate interaction models for waist circumference instead of BMI, we did not observe differences in the level of significance of the previously obtained results (P-int: 0.018 for *FTO*: P-int: 0.627 for *MC4R* and P-int: 0.021 for the aggregate score.)

Finally, taking into account that there is evidence to suggest [52] that dietary fiber could modify the association between the *FTO* rs9939609 and obesity risk and considering that folate intake is strongly correlated with fiber intake (rho=0.801; P<0.001 in this population), we have adjusted the effects of folate for total fiber intake (as continuous in g/d). After this additional adjustment in the multivariate model, the statistical interaction of the interaction term between folate intake and the *FTO* polymorphism or between folate and the aggregate score in determining fasting glucose concentrations in non-diabetic subjects did not change in significance level (P-int: 0.028 and P-int: 0.047, respectively).

## Discussion

In this study, in which type 2 diabetes cases and non-diabetic subjects did not differ in BMI, we found no statistically significant association between the *FTO* rs9939609 polymorphism and type 2 diabetes when analyzing the population as a whole. This result agrees with some previous studies in which no association with type 2 diabetes was reported [6,28-30]. However, in other investigations higher type 2 diabetes risk in carriers of the minor allele (obesity-risk allele) has been reported [13-17,20,21,23-29]. Among them there were many studies [1,23,27-29], including the first GWAs that detected the association between the *FTO* rs9939609 polymorphism and obesity risk [1], in which such association with type 2 diabetes disappears after adjusting for BMI, leading the authors to conclude that as the association between the *FTO* polymorphism and type 2 diabetes was mediated by BMI, the *FTO* is a susceptibility locus for obesity, but not for type 2 diabetes. However, in other reports [13-17,20,21] the association of the *FTO* minor allele with type 2 diabetes risk persisted even after adjustment for BMI increasing the evidence that the *FTO* can also be considered a diabetes-prone gene. However, some of these studies have been criticized for analyzing prevalent cases of type 2 diabetes and for differences of BMI between diabetic and non-diabetic subjects [24], recommending future case-control studies paired by BMI in order to better examine the independent effects. In the PREDIMED study, we fulfilled this requirement of having no differences in BMI between groups. This is a strength of our study and we were able to better analyze the effects of the *FTO* on type 2 diabetes more specifically.

Accordingly, the main finding and novelty of our results is that we have found that the association between the *FTO* rs9939609 polymorphism and type 2 diabetes depends on the diet consumed. Thus, when the dietary pattern departed from the traditional MedDiet (low-adherence to the MedDiet), the *FTO* rs9939609 was significantly associated with higher type 2 diabetes risk, while a good adherence to the MedDiet blunted this association. This gene-diet interaction was robust regardless of adjustment for BMI. Our results are supported by studies in mice in which a modulation by diet on the association of the fto gene with glucose intolerance has been reported [32]. As far as we know this is the first time that a significant interaction between the *FTO* rs9939609 and diet in determining type 2 diabetes has been reported in humans. Another study [53] concomitantly examined the effects of physical activity and caloric intake on the association between the *FTO* rs8050136 and diabetes in U.S. women, but found no statistically significant interaction. The small number of type 2 diabetes cases in that study [53] was a limitation.

There is increasing evidence that the MedDiet protects against type 2 diabetes [36,37], so it is not surprising that high adherence to this dietary pattern cancels the effects of greater genetic susceptibility to diabetes in *FTO* risk allele carriers. Such an interaction with diet might help explain the discrepancies in the published studies if those that did not find an association between the *FTO* rs9939609 polymorphism and diabetes risk [6,28-30] were enriched in subjects following a diet similar to high adherence to the MedDiet pattern, while studies that detected this association [13-17,21] dealt with populations with a less healthy dietary pattern, compatible with low adherence to the MedDiet.

One limitation of our study is that we analyzed prevalent cases of type 2 diabetes, as the incidence of diabetes in our cohort is still being compiled. Nevertheless, the dietary pattern of our study subjects was quite stable over time [44,54] and we did not detect differences in the adherence to the MedDiet depending on the duration of diabetes in this analysis. Similar results of no differences in diet were found in another study in Spain [35]. Thus, diabetes diagnosis did not change significantly the overall adherence to the MedDiet minimizing the reverse causation bias. Moreover, we have observed a similar protective effect of the MedDiet on type 2 diabetes risk when analyzing prevalent or incident type 2 diabetes cases in sub-samples of the PREDIMED study [37,54]. Likewise, in a Scandinavian population [14], the *FTO* rs9939609 was associated with both prevalent type 2 diabetes (OR 1.13; P<0.001) and the risk of developing incident type 2 diabetes (OR 1.16; P<0.001) having comparable results. Thus, although it is necessary to investigate the effect of the interaction between the level of adherence to the MedDiet and the *FTO* rs9939609 on incident type 2 diabetes cases in future studies, it is foreseeable that the results would be similar.

Just as for the *FTO* rs9939609, we found no associations of the *MC4R* rs17782313 with type 2 diabetes for the whole cohort despite a recent meta-analysis identifying the *MC4R* loci as a new loci related to type 2 diabetes in European populations [23]. Again, prior results from genetic association studies regarding this polymorphism and type 2 diabetes are discordant and sometimes vary after adjustment for BMI [2,18,19,22,25,26]. Although Qi et al [18] described a higher risk of type 2 diabetes in carriers of the minor allele, supporting preliminary data of Loos et al [2], Thomsen et al [25] in a large sample of Danish subjects did not find such association. Noticeably, we detected, for the *MC4R* rs17782313, a similar interaction with adherence to the MedDiet as for the *FTO* rs9939609, and this is also a relevant and novel finding of the present investigation. Moreover, when we analyzed the aggregate genetic score of the *FTO* and *MC4R* polymorphisms, we also observed an additive effect of these polymorphisms on the gene-diet interaction, thus strengthening our results.

Besides examining the genetic interactions with adherence to the MedDiet, we analyzed interactions with various macronutrients and food groups, but found none (not shown). This strengthens the notion that for dietary modulation the contribution of one food is not crucial, but it is rather the overall dietary pattern with various foods or nutrients synergizing among them that is important. Considering the significant gene-diet interaction results that we have obtained for the *FTO* and *MC4R* loci, it would be interesting in future studies to analyze this interaction for other polymorphisms previously associated with obesity and/or diabetes [55-58].

Finally regarding our secondary objective aimed on studying the role of folate intake in this gene-diet interaction, given that recent literature is highlighting the importance of epigenetics in insulin resistance and type 2 diabetes [42,59,60], we found interesting preliminary results that require confirmation in future studies. Although no interaction of folate intake with the genetic variants on type 2 diabetes was observed, we examined fasting glucose concentrations as a more dynamic diabetes-related trait and found a statistically significant interaction between the *FTO* rs9939609 polymorphism and folate intake in non-diabetic subjects. Thus, the *FTO* variant allele tended to be associated with higher fasting glucose concentrations when folate intake was low, but not when it was high. Currently, the *FTO* gene has been outlined as an important gene in which effects may be mediated through epigenetics [61]. A study reported that the CpG site in the first intron of the *FTO* gene was hypomethylated in type 2 diabetes cases relative to controls [60]. Folate is required for the synthesis of S-adenosyl methionine, which serves as a methyl donor for DNA methylation events; thereby folate availability may be crucial in the DNA methylation status [40]. The MedDiet is rich in folate and so one of the mechanisms underlying its protective effect against type 2 diabetes could be the influence of folate on DNA-methylation and fasting glucose. Some clinical trials have shown folic acid supplementation reduces insulin resistance [62]. Although it could be one of the mechanisms that may contribute to explaining the observed gene-diet interaction, we believe that it is not the only one and that more research has to be undertaken on this point. Although for *MC4R* we found no significant interaction with folate, there was a similar trend and, when analyzing the aggregate variable of both polymorphisms, the interaction term reached statistical significance, supporting additive effects. In this regard, there is a study in mice showing that diet might have an effect on the methylation status of the Mc4r gene [37]. However, our statistically significant results should be accepted with caution as folate intake may simply reflect a healthy dietary pattern and not a causal association with that micronutrient, given that, in our study, we did not carry out a methylation analysis to test this hypothesis. Moreover, although we have found a nominally significant interaction between the *FTO* polymorphism and the aggregate score and folate intake in determining fasting plasma glucose concentrations, we cannot rule out the possibility that, as this is a secondary hypothesis and we have not corrected the P-values for multiple comparisons, the association obtained represents a false positive result.

## Conclusion

In conclusion, we described for the first time a statistically significant gene-diet interaction of the *FTO* rs9939609 and *MC4R* rs17782313 with adherence to the MedDiet on type 2 diabetes. When adherence was low, the obesity risk alleles were associated with type 2 diabetes regardless of BMI, but more studies are needed to confirm this interaction. Although we have also found a statistically significant interaction with folate intake on fasting glucose that may help to explain in part this interaction, the potential mechanisms behind this interaction remain to be investigated in further studies.

## Competing interests

JSS is a non paid member of the Scientific Advisory Board of the International Nut Council, Reus, Spain. ER is a non paid member of the Scientific Advisory Committee of the California Walnut Commission, Sacramento, CA. The other authors have no competing interest affecting the conduct or reporting of the work submitted.

## Authors’ contributions

DC, RE, JMO, JVS, MAMG, ER, JSS, MIC, and LSM designed research; COA, JVS, EMA, MAMG, EGG, JSS, MF, MIC, ER, RE, FA, and LSM conducted research; JVS, MAMG, JSS, MIC, RE, FA, JL, LSM, EGG, MF, GST, XP, and MAM provided essential materials; DC, COA, JVS, OC analyzed data and performed statistical analysis; DC, JVS, and JMO wrote paper; DC and JVS had primary responsibility for final content. All authors made substantial contributions to conception and design, acquisition of data or analysis and interpretation of data, drafting the article or revising it critically for important intellectual content and approved the final version of the manuscript. All authors read and approved the final manuscript.
